# A pilot study evaluating the use of ABCD2 score in pre-hospital assessment of patients with suspected transient ischaemic attack: experience and lessons learned

**DOI:** 10.1186/s13231-016-0020-3

**Published:** 2016-08-20

**Authors:** Scott Munro, Sally Rodbard, Khalid Ali, Claire Horsfield, Wendy Knibb, Janet Holah, Ottilia Speirs, Tom Quinn

**Affiliations:** 1 South East Coast Ambulance Service NHS Foundation Trust, Banstead, Surrey SM7 2AS UK; 2 School of Health Sciences, Faculty of Health and Medical Sciences, University of Surrey, Guildford, Surrey GU2 7XH UK; 3 Brighton and Sussex Medical School, Brighton, BN2 5BE UK; 4 Frimley Health NHS Foundation Trust, Frimley, Surrey GU16 7UJ UK; 5 Faculty of Health, Social Care and Education, Kingston University and St George’s, University of London, London, SW17 0RE UK

**Keywords:** Transient ischaemic attack, ABCD2 score, Pre-hospital, Emergency medical services, Pilot study, Bureaucracy

## Abstract

**Background:**

Suspected transient ischaemic attack (TIA) is a common presentation to emergency medical services (EMS) in the United Kingdom (UK). Several EMS systems have adopted the ABCD2 score to aid pre-hospital risk stratification and decision-making on patient disposition, such as direct referral to an Emergency Department or specialist TIA clinic. However, the ABCD2 score, developed for hospital use, has not been validated for use in the pre-hospital context of EMS care.

**Methods:**

We conducted a pilot study to assess eligibility criteria, recruitment rates, protocol compliance, consent and follow-up procedures to inform the development of a definitive study to validate the ABCD2 tool in pre-hospital evaluation of patients with suspected TIA.

**Results:**

From 1st May–1st September 2013, nine patients with an EMS suspected diagnosis of TIA had the TIA diagnosis later confirmed by a specialist from five participating sites. This recruitment rate is comparable to stroke trials in the EMS setting. Bureaucratic obstacles and duplication of approval processes across participating sites took 13 months to resolve before recruitment commenced. Due to the initial difficulty in recruitment, a substantial amendment was approved to modify inclusion criteria, allowing patients with atrial fibrillation and/or taking anticoagulant therapy to participate in the study.

**Conclusions:**

It is possible to identify, recruit and follow up patients with suspected TIA in the EMS setting. Training large numbers of EMS staff is required as exposure to TIA patients is infrequent. Significant insight was gained into the complexity of NHS research governance mechanisms in the UK. This knowledge will facilitate the planning of a future adequately powered study to validate the ABCD2 tool in a pre-hospital setting.

**Electronic supplementary material:**

The online version of this article (doi:10.1186/s13231-016-0020-3) contains supplementary material, which is available to authorized users.

## Background

An estimated 20,000 people a year in the United Kingdom (UK) have a transient ischaemic attack (TIA), which is an important risk for an imminent stroke [1]. Patients who have ongoing symptoms on arrival at emergency medical services (EMS) are considered to have an acute stroke until proven otherwise. There are standard pathways in place for rapid referral of these patients, such as a hyperacute stroke unit (HASU) or an acute stroke unit.

Identifying patients with TIA who are at high risk of a subsequent stroke is crucial as the period following a TIA is an opportunity to provide interventions to reduce the risk of a future stroke. Following a TIA, patients should receive specialist evaluation and management of their risk factors. The quality standard for TIA published by the UK National Institute for Health and Care Excellence (NICE) states that ‘People seen by ambulance staff outside of hospital, who have sudden onset of neurological symptoms, [are] screened using a validated tool to diagnose stroke or TIA’ [2]. The ABCD2 score, developed for assessing stroke risk in patients with suspected TIA, has been widely implemented in a range of settings [hospitals, primary care, Emergency departments (ED) and some EMS systems]. However it has not yet been prospectively validated in the context of pre-hospital care [3–6].

ABCD2 allocates points for key clinical and vascular risk variables with total ABCD scores ranging from 0 to 7 points. In many guidelines total scores dichotomize between high (ABCD2 ≥ 4) and low (ABCD2 < 4) stroke risk, higher risk patients being targeted for specialist assessment within 24 h. A comparison of ‘first-contact’ healthcare professionals and stroke physicians has, however, raised concerns about the validity of non-specialist assessment of ABCD2 scores [7]. Moreover, a recent UK National Institute of Health Research (NIHR) funded study concluded that it was not cost effective to encourage the use of EMS to expedite rapid treatment of TIAs [8]. Currently there is no validated tool for TIA risk-assessment for use in the EMS setting.

The UK national guidelines for pre-hospital care do not recommend use of the ABCD2 by EMS due to lack of supporting evidence [9, 10]. Therefore the role of ABCD2 as a risk stratification tool in the EMS setting requires further evaluation.

The objective of this pilot study is to assess eligibility criteria, recruitment rates, protocol compliance, consent and follow-up procedures to inform the development of a definitive study to validate the ABCD2 as a tool for identifying patients with suspected TIA, assessed by EMS staff in pre-hospital settings, and follow them, for TIA or stroke recurrence in the following 7 and 90 days.

## Methods

### Study design and setting

We performed a pilot, prospective, multi-centered observational study undertaken in two counties served by a large regional EMS in the UK. The EMS serves a total population of approximately 4.5 million, employs approximately 1140 paramedics and emergency medical technicians (EMTs) and received 862,446 emergency calls in 2013/2014, of which 8940 (1 %) were coded by the Emergency Operations Centre dispatch computer as suspected stroke/TIA. The study included five receiving hospital sites.

### Staff training

A standardized training package (Additional file 1) for the study was developed by the study team in collaboration with EMS educators. This included an update on stroke and TIAs and introduced the concept of risk scoring, using the ABCD2 tool, and study procedures and documentation. Training was delivered by EMS clinical supervisory staff, all experienced paramedics, to 146 EMS personnel (paramedics and emergency medical technicians) from one county, representing 58 % of staff eligible to be trained. A ‘cascade’ approach to training was used as this is the standard method employed by the participating EMS. Cascade or ‘train-the-trainer’ approaches involve training a small group who then pass on what they have learnt to the rest of the workforce. Participating staff completed a Learning Validation Form confirming they had read and understood the training material and would adhere to study procedures, and a central log of all staff trained was maintained by the study co-ordinator. Staff in a neighbouring county (part of the same EMS) were not trained in the study procedures, but were asked to provide suspected TIA patients with a study pack, to help assess whether additional training resulted in increased patient identification and recruitment.

We collected data on whether EMS staff were able to recruit patients according to study inclusion and exclusion criteria, whether the EMS diagnosis of TIA was confirmed subsequently by a hospital specialist, and whether patients would consent for follow up. We also collected data on the number of patients who had suspected TIA at the time of EMS assessment but were not recruited to help in planning the main study. We compared recruitment rates for the county where EMS staff received additional training compared with the county where no additional training was provided.

### Inclusion/exclusion criteria

Inclusion/exclusion criteria were developed with the advice of a multidisciplinary steering group comprising stroke physicians, EMS staff and patient representatives. All patients aged 18 years or over with symptoms suggestive of TIA and could speak and understand English were eligible for the study. Patients were excluded from the study in the following circumstances:

- Continuing stroke symptoms at EMS assessment.
- Prior stroke or TIA.
- History suggestive of ‘crescendo’ TIAs.
- Anticoagulant medication.
- Atrial fibrillation.
- Prosthetic heart valves.
- Under the age of 40 with likely TIA and neck pain (suggestive of dissection).
- Otherwise medically unstable.
- Active current malignancy.
- Known or suspected pregnancy.

Patients were assessed by EMS staff and if they had a suspected TIA and met the study inclusion criteria, were provided with a pack containing a Patient Information Sheet and Consent Form for follow-up, which they were requested to return in a stamped addressed envelope to the study co-ordinator. Study packs were also available in participating hospital Emergency Departments and specialist TIA clinics. EMS staff completed a standard study proforma (Additional file 1), which included baseline demographic and clinical data, and calculated the ABCD2 score. As agreed with the Steering Group and Ethics Committee, the ABCD2 score was not used to determine risk or guide patient disposition as the tool has not been validated in this setting. Hence all patients will have received standard care and been taken to hospital for further assessment, unless they declined transport. Patients in the county where EMS staff were not provided additional training followed the standard EMS pathway into hospital (Additional file 1).

### Hospital care and follow-up

**Fig. 1:**
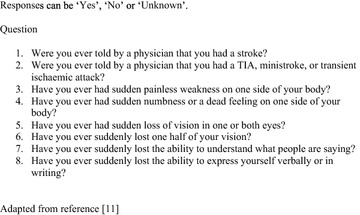
Questionnaire for validating stroke-free status

Patients underwent standard evaluation in hospital according to local protocols. Patients who had the diagnosis of TIA confirmed by a hospital specialist and who had returned a signed consent form were followed up by a structured telephone interview at 7 and 90 days by a researcher trained in the use of the Questionnaire for Validating Stroke-Free Status (QVSFS) (Fig. 1) [11]. The interviewer was blinded to patient characteristics and ABCD2 scores.

## Results

**Fig. 2:**
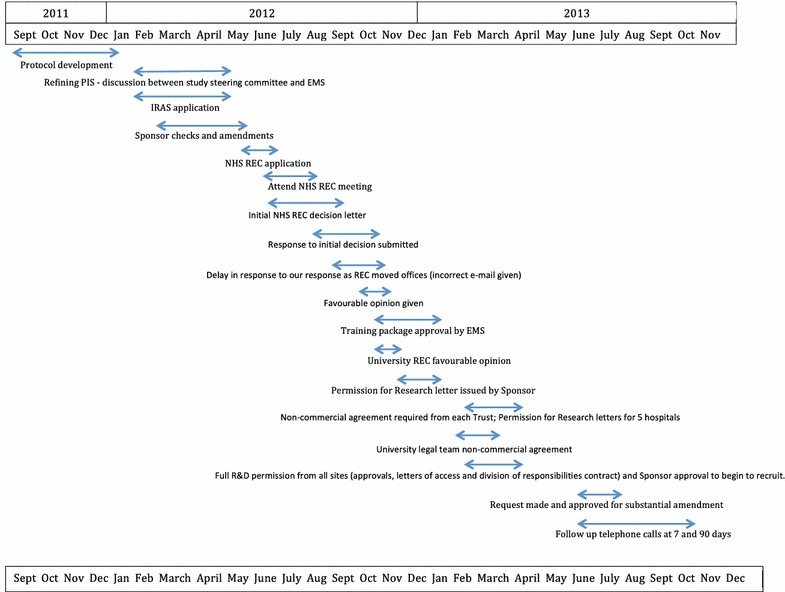
Study approval timeline

Recruitment to this observational pilot study was delayed while the study Sponsor, two Ethics Committees, the EMS Research Committee and five hospital Research Offices individually reviewed and requested revisions to the study protocol and other documentation (Fig. 2). Following four of the hospitals issuing ‘Permission for Research’ letters, the fifth hospital’s Research Office required a non-commercial trial agreement be signed by all collaborating sites, further delaying the process. Thirteen months lapsed from the date of initial submission to the NHS Ethics Committee to all final approvals being in place.

**Fig. 3:**
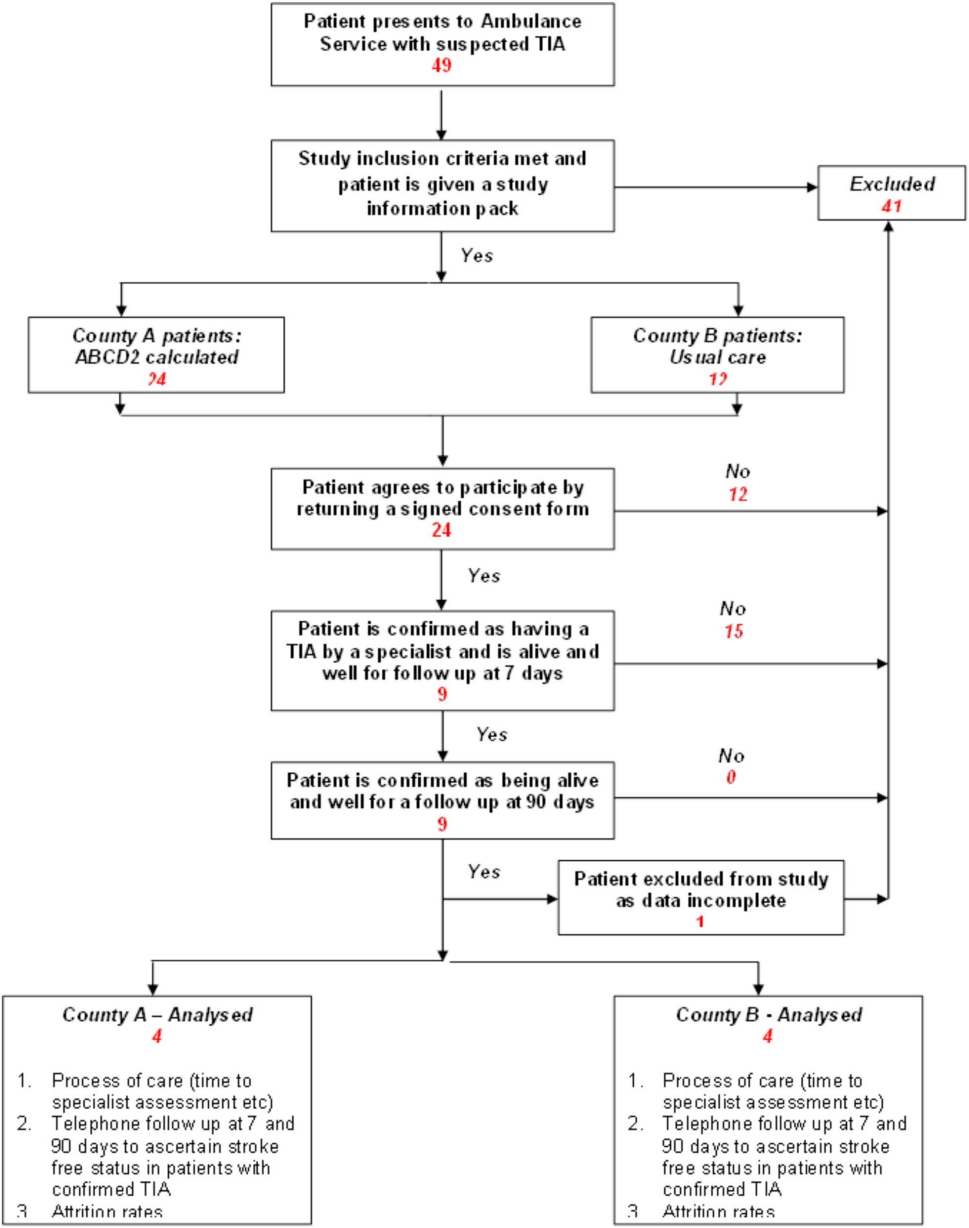
Study flow chart

From 1st May–1st September 2013, 49 suspected TIA patients were screened by EMS staff trained in study procedures across two counties nine of these 49 patients had confirmed TIA diagnosis following specialist assessment, and gave consent for follow up. All patients were known to be alive at 7 and 90-day follow-up. However, one patient was subsequently excluded due to incomplete EMS data, leaving 8 patients enrolled in the study (see Fig. 3). Patient characteristics are shown in Table 1. The number of recruited patients was the same, namely four patients in each county.

**Table 1:** Baseline characteristics

	County A	County B
Male n (%)	2 (50)	4 (100)
Age, mean (range)	68 (54–81)	79 (71–91)
White n (%)	3 (75)	4 (100)
ABCD2 score by EMS n (%)		
Age ≥ 60	3 (75)	4 (100)
BP ≥ 140/90 mmHg	4 (100)	4 (100)
Clinical features		
Unilateral weakness	1 (25)	n/a
Speech disturbance without weakness	1 (50)	n/a
Neither	1 (25)	n/a
Duration ≥ 60 min.	1 (25)	n/a
Diabetes	1 (50)	1 (25)
EMS ABCD2 ≥ 4	4 (100)	n/a
Specialist ABCD2 ≥ 4	2 (50)	n/a
Past medical history		
Hypertension	2 (50)	1 (25)
Atrial fibrillation	0 (0)	1 (25)
Ischaemic heart disease	0 (0)	0 (0)
Carotid stenosis	0 (0)	0 (0)
Peripheral vascular disease	0 (0)	0 (0)
Smoker	0 (0)	0 (0)
Prosthetic heart valve	0 (0)	0 (0)
Medication		
Clopidogrel	1 (25)	0 (0)
Dipyridamole	0 (0)	0 (0)
Time to specialist within target	3 (75)	4 (100)
Investigations		
MRI scan	1 (25)	1 (25)
Carotid Doppler	3 (75)	3 (75)
12 lead ECG	3 (75)	2 (50)
CT brain	3 (75)	3 (75)
Follow up stroke free status		
7 Days	3 (75)	4 (100)
90 Days	3 (75)	3 (75)

Initial recruitment was poor with no patients being recruited in the 1st month. Analysis of EMS records revealed that potential patients were not being recruited due to the stringent exclusion criteria. A substantial amendment was approved by the Steering Group and Ethics Committees to remove three exclusion criteria (previous TIA, atrial fibrillation and anticoagulation therapy), and to extend recruitment by 1 month. Reasons for non-recruitment of patients where EMS staff suspected TIA, by county, for each month of the study before and after protocol amendments are shown in Table 2.

**Table 2:** Reasons patients were not recruited to the pilot study

Reason	Month 1		Month 2		Month 3		Month 4	
County	A	B	A	B	A	B	A	B
Ongoing symptoms	13	4	12	12	3	2	1/13	2
Prior stroke/TIA^a^	14	9	21	9	3	2	1/0^b^	1
Anticoagulants^a^	11	4	7	6	5	2	3/1	1
Atrial fibrillation^a^	3	7	1	1	0	0	0/0	0
No consent returned	0	0	0	0	0	0	0/0	0
Patient declined	1	0	0	0	0	0	0/0	0
Not conveyed	4	4	0	1	2	1	0/0	0
Crew not trained	10	0	0	0	1	0	0/0	0
Not known	3	4	5	7	0	0	7/10	1

County A—EMS crews trained, County B—EMS crews not trained, merely handing study packs to patients

^a^Exclusion criteria subject to substantial protocol amendment approved by ethics committee

^b^Denotes numbers before and after study protocol amended to relax exclusion criteria

### Sponsor audit

The pilot study underwent a random, announced, research governance audit by the Sponsor over a 2 day period in February 2014 (recruitment had, as planned, ended on 1st September 2013). This was a useful exercise in confirming compliance with the requirements of the National Health Service (NHS) Research Governance Framework. However this audit was conducted according to a Sponsor proforma designed for clinical trials of investigational medical products (CTIMP). Subsequent feedback from the audit team to the study team recommended that a Delegation of Responsibilities Log for EMS staff was required in addition to the existing training log held by the study co-ordinator, and signed curricula vitae were required from all participating EMS staff. These may be considered as excessive requirements given that EMS staff were merely handing patients an envelope containing study information and not obtaining consent or initiating an intervention. The chief investigator and study co-ordinator had current certification in Good Clinical Practice which is a standard designed to meet regulatory requirements for CTIMP studies rather than pilot, non-interventional studies such as ours. The team were otherwise commended on the quality of the study site file.

## Discussion

Pilot studies focus on the processes of the main study rather than seeking to determine the utility of an intervention and we report our findings on that basis, together with our experience, in this pre-hospital setting of UK NHS research governance processes which are primarily designed for hospital trials of investigational medical products.

### Regulatory burden

Each participating hospital had a different approach to granting approval for the study. In this pilot study, each hospital Research Office reviewed the protocol and study documentation, duplicating efforts already undertaken by the study Sponsor, and leading to an overall delay of 31 months before patient recruitment could commence. Snooks et al. [12] and Thompson et al. [13] have previously highlighted the ‘stifling’ bureaucratic structures and processes for research governance that lead to barriers to undertaking medical research. It should be noted that when the substantial amendment was requested the study passed through the review process with relative ease, reducing any further delay once recruitment was underway. The new UK Health Research Authority (introduced in late 2011), which has responsibility for oversight of ethical and governance processes for research in the NHS, is consulting on a new regulatory framework, which will lead to a more proportionate approach to low risk studies such as ours.

### Study processes

Our pilot study demonstrates that it is possible to conduct a prospective observational study of EMS use of the ABCD2 score in suspected TIA patients. The research-trained paramedics and technicians were able to identify suitable patients, complete initial assessments and follow study procedures, and the study team was able to obtain consent and complete follow up.

### Training

The dissemination of training for paramedics and technicians using cascade training is standard for introduction of new procedures across a busy UK EMS, reflecting the challenges of reaching large numbers of staff, distributed across large geographical areas and working shift patterns in the mobile, high pressure and unpredictable prehospital environment. This is a very different environment from the outpatient clinic or hospital ward, where arguably communication is simpler by comparison.

We trained almost two-thirds of eligible EMS staff in one county, but since only 49 TIA patients were recorded across the two counties during a 4 month period, it is likely that exposure of an individual paramedic or EMT to a patient with TIA is infrequent, in common with other conditions such as cardiac arrest where on average an individual paramedic will encounter a single patient in a year, as reported by a recent UK trial [14]. In a pilot randomized trial involving EMS recruitment of stroke patients, 14 patients were recruited over 14 months, a rate similar to our experience [15]. Our study experience and recruitment figures suggest that for a future study to recruit enough patients for an adequately powered study, significant efforts are needed to train very large numbers of EMS staff. We did not survey EMS staff about their perceptions of the research process for our study. Other UK investigators surveyed paramedics engagement in a feasibility trial in an ultra-acute stroke setting, and identified lack of institutional support for research, a learning curve, and (as we highlight) rarity of eligible patients and lack of time for training as important barriers to study success in the EMS environment. EMS research is a relatively recent phenomenon, and paramedics have mixed views on their responsibilities: a survey in the United States indicated that paramedics were interested in taking part in research, but that only 38 % of them would want the right not to participate [16]. This requires further study and education for UK EMS personnel around their professional responsibilities to engage with research.

## Conclusion

It is possible to identify, recruit and follow up patients with suspected TIA in the EMS setting. Training large numbers of EMS staff is required as exposure to TIA patients is infrequent. Significant insight was gained into the complexity of NHS research governance mechanisms in the UK. This knowledge will facilitate the planning of a future adequately powered study to validate the ABCD2 tool in a pre-hospital setting.

## Additional file

10.1186/s13231-016-0020-3 Evaluation of the ABCD2 Score in pre-hospital assessment of Patients with suspected Transient Ischaemic Attack: pilot study.

## Abbreviations

ABCD2: age, blood pressure, clinical features, duration of transient ischaemic attack, and presence of diabetes
CTIMP: clinical trial of an investigational medicinal product
ED: emergency department
EMS: emergency medical services
EMT: emergency medical technician
HASU: hyper acute stroke unit
NHS: National Health Service
NICE: National Institute for Health and Care Excellence
NIHR: National Institute for Health Research
QVSFS: questionnaire for validating stroke-free status
TIA: transient ischaemic attack
UK: United Kingdom
