# Supplementary material for: A pilot study evaluating the use of ABCD2 score in pre-hospital assessment of patients with suspected transient ischaemic attack: experience and lessons learned
Source: Exp Transl Stroke Med. 2016 Aug 20;8:6. doi: 10.1186/s13231-016-0020-3 (PMC4992554; doi:10.1186/s13231-016-0020-3)
Supplement: Supplementary file 1 — 10.1186/s13231-016-0020-3 Evaluation of the ABCD2 Score in pre-hospital assessment of Patients with suspected Transient Ischaemic Attack: pilot study. [file 13231_2016_20_MOESM1_ESM.pdf]

South East Coast Ambulance Service NHS
NHS Foundation Trust

UNIVERSITY OF SURREY

Evaluation of the ABCD2 Score in pre-hospital assessment of Patients with suspected Transient Ischaemic Attack: pilot study

Cascade training package for Paramedics and Technicians

Individual learning material

Notes:

These learning materials are aimed at ambulance clinicians working in Surrey. However, they may also be issued to Emergency Care Support Workers and Community First Responders who may benefit from the information contained when assisting their colleagues.

Guidelines for study

These learning materials have the following purposes:

- They are designed to facilitate pre-learning for paramedics and technicians and other relevant clinical staff who are taking part in this pilot study in Surrey.
- They should be retained as reference material by paramedics and technicians taking part in the pilot study.
- They may be used as a reference guide by all ambulance staff that assist paramedics and technicians in the management of patients who are eligible for inclusion in this pilot study.

This study guide can be retained as evidence of your professional development.

Learning outcomes

On completion of this learning material, the ambulance clinician should be able to:

- Describe the purpose of the TIA pilot study.
- Describe the assessment and treatment procedures for patients with suspected transient ischaemic attack (TIA).
- When attending to an eligible patient, be able to apply the study protocol.

Glossary or Terms

ABCD2	a scoring system designed to help assess the risk of stroke after a transient ischaemic attack, currently validated for use in hospital.
Cohort study	a study design which identifies a group of people and follows them over a period of time to see how their exposures affect their outcomes.
Crescendo TIA	two or more TIAs within 1 week.
Exclusion criteria	the medical or social standards determining whether a person may not be allowed to enter a research study.
Haemodynamic instability	a state requiring pharmacologic or mechanical support to maintain a normal blood pressure or adequate cardiac output.
Inclusion criteria	the medical or social standards determining whether a person may be allowed to enter a research study.
JRCALC	Joint Royal Colleges Ambulance Liaison Committee. A national committee established to provide robust clinical advice to ambulance services. Responsible for development of UK national ambulance guidelines.
National stroke strategy	the Department of Health's 10 year strategy, intended to provide a quality framework to secure improvements to stroke services, to provide guidance and support to commissioners and strategic health authorities and social care, and inform the expectations of patients and their families by providing a guide to high quality health/social care services.
NICE guideline CG68	national clinical guideline for diagnosis and initial management of acute stroke and transient ischaemic attack (TIA).
Specialist assessment	evaluation by a person highly skilled in a specific and restricted field (e.g. a consultant or specialist nurse).
Stroke	a clinical syndrome consisting of rapidly developing clinical signs of focal (or global in case of coma) disturbance of cerebral function

lasting more than 24 hours or leading to death with no apparent cause other than a vascular origin.

Stroke networks — encompass the whole stroke pathway by connecting different organisations and teams involved along the patients journey, so individuals experience co-ordinated management from the first contact which extends to lifelong support as a stroke survivor. Networks involve stroke survivors and carers as active partners in coordinating and supporting service development.

TIA — Transient Ischaemic Attack. Symptoms and signs of acute stroke resolving up to 24 hours after onset.

CONTENTS

1. Background 6

2. Study aims 7

3. Study participants 8

4. Eligibility 8

5. Consent 9

6. ABCD2 score 10

7. The referral pathway 10

8. Re-cap on TIA's 12

9. References 14

Appendices

A. TIA referral form (within study envelope) 16
B. Study flow diagram (within study envelope) 18
C. Patient information sheet (in red patient envelope) 19
D. Patient consent form (in red patient envelope) 22

1. Background

a. Burden of stroke and transient ischaemic attack (TIA)

In recent years stroke has come to be regarded as a medical emergency, with time-dependent treatments such as thrombolytic therapy which have been shown to improve outcomes, principally through reduction in disability. Ambulance services have been recognised as an important component of the 'Chain of recovery' following stroke and as ambulance clinicians we are trained in recognition of acute stroke using validated tools such as the Face Arm Speech Test (FAST).

There is as yet no assessment tool for TIA validated for use in the ambulance setting. The risk of a patient with TIA developing a stroke is high and symptoms should always be taken seriously. One in eight patients with a TIA will go on to have a stroke within 2 days (table 1.1). However, anecdotal evidence from ambulance staff suggests that a significant proportion of patients in whom TIA is suspected decline transport to hospital as they 'feel better'.

Table 1.1

ABCD2 SCORE	RISK OF STROKE AT 2 DAYS
0 - 3	**1%**
4 - 5	**4%**
6 - 7	**8%**

Timely assessment and treatment of patients with suspected TIA is a Quality Marker in the National Stroke Strategy and recommended in NICE guidelines. Identifying patients with suspected TIA who are at high risk of stroke is an opportunity to prevent stroke, and early initiation of treatment following a TIA can reduce the risk of stroke by up to 80%, yet current evidence suggest the time delay between presentation of symptoms to specialist assessment to be between 11 and 15 days. NICE Guidelines recommend that those TIA patients deemed of high risk of having a stroke should be seen by a specialist within 24 hours, and within 7 days if at low risk.

The ABCD2 score, developed in Oxford and California, calculates the risk of stroke from 5 simple criteria: **A**ge, **Bl**ood pressure, **Cl**inical presentation, **D**uration and **D**iabetes, and is based on clinical characteristics detected at the time of initial assessment. The score is

designed to enable clinicians to assess the risk of a patient proceeding to full stroke. It has been implemented in a range of settings (hospitals, primary care, A+E's and some ambulance services) but has not yet been validated in the context of pre-hospital care and is therefore not recommended in current guidelines for pre-hospital care. Moreover, a recent National Institute for Health Research, Service, Delivery and Organisation (NIHR SDO) report , concluded that it was not cost effective to encourage use of emergency ambulances to expedite rapid treatment of patients with suspected TIA. Another high quality, multicentre study attempted to validate the ABCD2 score in the Emergency Department setting, but found the score to be inaccurate as a predictor of imminent stroke. Alongside similar studies, data suggest ABCD2 performs poorly in the emergencysetting. There are no high quality prospective studies evaluating the ABCD2 score in the pre-hospital setting in the published literature to our knowledge.

b. Current UK ambulance guidelines for TIA assessment and care

UK guidelines of relevance include the National Stroke Strategy, NICE guideline CG68 and the NICE Quality Standard, as well as JRCALC.

The NICE Quality standard states:

> *People seen by ambulance staff outside hospital, who have sudden onset of neurological symptoms, are screened using a validated tool to diagnose stroke or transient ischaemic attack (TIA).*

JRCALC guidelines recommend all patients suspected of TIA are taken to hospital. Despite its introduction into some ambulance services, the national guidelines for pre-hospital care do not recommend use of the ABCD2 score at this time due to the lack of evidence.

2. Study aims

The objective of this study is to externally validate the ABCD2 score as a tool for identifying patients with suspected TIA, assessed by ambulance staff in the pre-hospital setting, who are at high risk of stroke within 7 and 90 days. We will introduce the ABCD2 score into our pre-hospital assessment for Surrey patients for a 3 month period and assess how well it performs . We will also collect data on whether ABCD2 performed in the pre-hospital setting makes a difference to the time taken for a patient to see a TIA specialist.

3. Study participants

All patients aged 18 years or over attended by study-trained SECAmb ambulance personnel (paramedics and technicians) following a 999 call, in which the attending ambulance clinician suspects a diagnosis of TIA, will be enrolled.

This study is for Chertsey and Guildford ODA's only, and the hospitals participating are St.Peter's, Frimley Park and the Royal Surrey. Patients taken to other hospitals are not eligible for this study.

All suspected TIA patients will undergo standard pre-hospital assessment of their presenting complaint, past medical history and baseline clinical assessment (vital signs, ECG, pulse oximetry, Glasgow Coma Score, FAST test, blood sugar) in accordance with standard SECAmb guidelines. Patients will also have ABCD2 calculated by ambulance staff.

4. Eligibility

All patients to be entered into the study must have met the inclusion criteria . If they do not meet the criteria, then they are not eligible to be in the study, there is no need to calculate an ABCD2 score and standard care applies.

a. Inclusion criteria

- Aged 18 years or over
- Symptoms suggestive of TIA
- Able to speak and understand English (patients are to be followed up at a later date via a telephone call so English is essential).

b. Exclusion criteria

- Continuing stroke-like symptoms at time of ambulance assessment
- Known prior stroke or TIA at any time before this event
- Patients with 'crescendo' TIAs*
- TIA and on anticoagulants

- TIA and medically unstable
- TIA plus A.F
- TIA plus prosthetic valve
- Young patients (under 40) with likely TIA and neck pain
- TIA and malignancy
- TIA and pregnancy

* Someone who has had more than two TIA's in one week are termed as having crescendo TIA.

5. <u>Consent</u>

This study has a favourable ethical opinion from the the National Research Ethics Service South East Coast – Surrey, and from the University of Surrey Ethics Committee, and has NHS Research approval from SECAmb. (Ethics reference number 12/LO/1025)

As this is a research study each patient will need to consent to be a participant. Full informed consent will not be possible in a patient in the acute phase of pre-hospital care where time is limited and patients are likely to be anxious and distressed following a 999 call.

An information envelope for the patient has been developed. This contains all the information about the study as well as a consent form which they can send back in the stamped addressed envelope provided. We will ask you to give this envelope to your patient at an opportune point before handover at hospital, or give it to the handover nurse if judged appropriate.

6. The ABCD2 score

The risk of stroke following TIA can be calculated from 5 simple criteria, which together are termed the ABCD2 score.

ABCD2 COMPONENTS	CRITERIA	POINTS
AGE	Aged over 60 years	1
BP	Hypertension (Systolic >140 and/or diastolic >90mmHg)	1
CLINICAL FEATURES	Unilateral weakness	2
	Speech disturbance without weakness	1
DURATION	Over 60 Mins	2
	10 – 59 mins	1
DIABETES	YES	1

If the patient has an ABCD2 score of 4 or greater, they are deemed to be at high risk. If the patient presents with a score of 3 or less, they are deemed to be at low risk.

Please note that since the ABCD2 has not been validated in the pre-hospital setting (the reason we are doing the present study) ambulance clincians must not use the score to inform their decision making.

7. The referral pathway

a. Assessing the patient

If you suspect your patient to have suffered a TIA (i.e. stroke-like symptoms that have completely resolved), having considered differential diagnoses, then it is important to complete a full set of observations as you would do normally. This includes blood glucose (to rule out hypoglycaemia) and a 12 lead ECG (to rule out arrhythmias). Technicians should

perform a 12 lead ECG but are not expected to interpret it or identify arrhythmias unless they already do this. Where the patient has continuing symptoms suggestive of acute stroke at the time of assessment, then they should receive standard care according to our agreed stroke pathway.

A study pack has been developed, one per patient, containing a study flow diagram, a study form and the patient information envelope. The study form needs to be completed for every patient in whom you suspect TIA. **All eligible patients should be included and given the information envelope.**

.

Follow all 3 steps within the study form and then fill out your PCR as you would normally, also completing the ABCD2 score, which can be put in the dedicated box in the stroke section. Make sure that in your free text box you describe your patient's clinical features (which should have resolved) and document the duration of their symptoms. If your patient is not eligible, see below.

All patients, whether their score is high or low risk, should be taken to the A+E as per normal practice. If the setting allows you can briefly discuss this study with your patient and give them the information envelope (found inside your study pack). Their treatment will not change because of this intervention, the purpose of the information envelope is to tell them about the project and to invite them to complete and return the consent form giving permission for a follow up phone call at 7 and 90 days to check on the status of their health. We are not gaining consent to calculate the ABCD2 score. If the patient reads, signs and sends back the stamped envelope then consent will be assumed by the research team.

b. Non-eligible patients

If you suspect TIA, but after completing the TIA form find the patient is not eligible for this study, continue your care as per normal. It would be useful to the study to document the reason on your PCR for why the patient was not t eligible. Keep the completed study form with your PCR and hand it in at the end of the day on station. There is no need to complete an ABCD2 score as this patient is not eligible to be entered into the study.

NB Exclusion criteria question 7 on the study form is 'to confirm that your patient is not in A.F'. If you are a technician and are <u>not</u> normally expected to interpret or identify arrhythmias

then you should tick no to this question and your patient is not eligible to be in the study (follow the guidelines above).

c. Non-conveyance

Do your best to encourage your patient to be taken to hospital. Should they decline transport, complete your PCR as per normal, document their refusal, complete the disclaimer and keep the study form to hand in at the end of the day on station with your PCR.

If your patient declines transport to hospital then, as per normal practice, ensure your patient understands why you want to take them and what to do if their condition deteriorates. Keep in mind your patients capacity and the need for you to refer safely. For guidance, please refer to the Trust's Non-Conveyance Guidelines.

See appendix B for pathway flow diagram.

8. Re-cap on TIA

a. TIA

Transient ischaemic attack is defined as stroke symptoms and signs that resolve within 24 hours. The symptoms of a TIA usually resolve within minutes or a few hours at most, and anyone with continuing neurological signs when assessed should be assumed in the first instance to have had a stroke.

The risk of a patient with TIA developing a stroke is high and symptoms should always be taken seriously.

b. Signs and symptoms

Signs and symptoms of a TIA are those of a stroke, which include:

- numbness
- weakness or paralysis
- slurred speech
- blurred vision
- confusion
- severe headache

c. Assessment – Face, Arm, Speech, Test (FAST)

Face – did they have any weakness? Did their face fall on one side. Could they smile?
Arm – did they have any weakness? Could they raise both their arms and leave them there?
Speech – did they have problems? Was their speech slurred?

If the patient had a deficit in any one of the three domains (which have now resolved), it is sufficient for the patient to be identified as having been 'FAST positive' and TIA presumed.

9. References

1. Department of Health. National Stroke Strategy http://www.dh.gov.uk/en/Publicationsandstatistics/Publications/PublicationsPolicyAndGuidance/DH_081062 (accessed 11 January 2012)

2. Giles MF, Rothwell PM. (2010). Systematic review and pooled analysis of published and unpublished validations of the ABCD and ABCD2 transient ischemic attack risk scores. *Stroke*.41:667-73.

3. Hankey, G. & Warlow, C. (1994) *Transient ischaemic attacks of the brain and eye.* London: WB Saunders.

4. Harbison J, Hossain O, Jenkinson D, Davis J, Louw SL, Ford G. Diagnostic accuracy of stroke referrals from primary care, emergency room physicians, and ambulance staff using the face arm speech test.*Stroke*. 2003;34:71-76.

5. Intercollegiate Stroke Working Party. *National clinical guideline for stroke*, 3rd edition. London: Royal College of Physicians, 2008.

6. Johnston C, Rothwell P, Nguyen-Huynh MN, Giles M, Elkins J, Bernstein A, Sidney S. Validation and refinement of scores to predict very early stroke risk after transient ischemic attack. *Lancet*.2007;369:283-92

7. Joint Royal Colleges Ambulance Liaison Committee (2009) Stroke/Transient ischaemic attack – updated guidance. http://www2.warwick.ac.uk/fac/med/research/hsri/emergencycare/prehospitalcare/jrcalcstakeholderwebsite/a-z/specific/stroke/ (accessed 11 January 2012)

8. Josephson A, Sidney S, Pham, TN, Bernstein AL, Johnston C. Higher $ABCD_2$ score predicts patients most likely to have true transient ischemic attack. *Stroke.*2008;39:3096-3098.

9. Mant J, Ryan R, McManus R, Fletcher K, Kandiyali, R, Barton P, Bryan S, Stevens A, Ford G, Rothwell P. What is the optimum model of service delivery for transient ischemic attack? April 2008.Report for NCCSDO.

10. Meschia JF, Brott TG, Chukwudelunzu FE, Hardy J, Brown RD Jnr, Meissner I, Hall LJ, Atkinson EJ, O'Brien PC. Verifying the stroke-free phenotype by structured telephone interview. 2000. *Stroke*. 31:1076-80.National Institute for Health and Clinical Excellence. (2008) Stroke: Diagnosis and initial management of acute stroke and transient ischaemic attack (TIA). NICE CG 68. http://www.nice.org.uk/guidance/CG68/NICEGuidance/doc (accessed 11 January 2012)

11. National Institute for Health and Clinical Excellence. (2011) http://pathways.nice.org.uk/pathways/stroke#content=view-info%3Aquality-standards-stroke-quality-standard (accessed 11 January 2012)

12. National Institute for Health Research Evaluation. Trials and Studies Co-ordinating Centre (NETSCC). Feasibility and pilot studies. http://www.netscc.ac.uk/glossary/#glos6 (accessed 11 January 2012)

13. National Research Ethics Service guidance on adults lacking Capacity to consent to research. http://www.nres.npsa.nhs.uk/news-and-publications/news/release-of-adults-lacking-capacity-toolkit/ (accessed February 2012).

14. Nor AM, McAllister C, Louw SJ, Dyker AG, Davis M, Jenkinson D, Ford GA. Agreement between ambulance paramedic-and-physician-recorded neurological signs with face arm speech test (FAST) in acute stroke patients.*Stroke*. 2004; 35: 1355-59.

15. Perry J, Sharma M, Sivilotti, MLA, Sutherland J, Symington C, Worster A, Emond M, Stotts G, Jin AY, Oczkowski WJ, Sahlas DJ, Murray HE, MacKey J, Verreault S, Wells GA, Stiell IG. Prospective validation of the ABCD2 score for patients in the emergency department with transient ischemic attack. *CMAJ*. 2011;10:1503.

16. Quinn T, Cameron AC, Dawson J, Lees K, Walters MR. ABCD2 scores and prediction of noncerebrovascular diagnoses in an outpatient population.*Stroke*.2009:40:749-753.

17. 'Stroke is a Medical Emergency' says The Stroke Association. http://www.stroke.org.uk/media_centre/press_releases/stroke_is_a.html (accessed 11 January 2012).

18. Wong J, Fotherby M, Eveson D. Comparison of ABCD2 scoring between first healthcare-contact and stroke-specialist physicians for transient ischaemic attack in a rapid-access clinic. *Age Ageing*. 2012:41:115-8.

South East Coast Ambulance Service NHS
NHS Foundation Trust

UNIVERSITY OF SURREY

Appendix A

Evaluation of the ABCD2 Score in pre-hospital assessment of Patients with TIA

STUDY FORM - SURREY

Patient name: ____

D.O.B: (dd/mm/yy) __/___/___ Job number: ______________________

Todays date (dd/mm/yy): __/___/______ Tel number: ______________________

STEP 1:

**Inclusion/exclusion criteria**	**Y**	**N**
Has your patient had symptoms of a stroke which are now completely resolved?	☐	☐
Can you confirm that your patient has not suffered a previous TIA or stroke?	☐	☐
Can you confirm that your patient is over 18 years old?	☐	☐
Can you confirm that your patient is not on anticoagulants?	☐	☐
Can you confirm that your patient speaks and understands English?	☐	☐
Can you confirm that your patient has not had 2 or more TIA's in the last week?	☐	☐
Can you confirm that your patient is not in AF?	☐	☐
Can you confirm that your patient does not have a prosthetic valve?	☐	☐
Can you confirm that your patient is not under 40 and suffering with neck pain?	☐	☐
Can you confirm that your patient does not have any form of malignancy?	☐	☐
Can you confirm that your patient is not medically unstable?	☐	☐
Can you confirm that your patient is not pregnant?	☐	☐
Can you confirm your patient is not hypoglycaemic?	☐	☐

- If all answers are yes, move on to step 2.
- If any answers are no, this patient is not eligible for this study. Do not move on to step 2. Follow your usual care. Please document this on your PCR, and keep this form in with your PCR to hand in at the end of the day on station.

STEP 2:

ABCD2 score (please circle your points)

ABCD2	CRITERIA	POINTS
**AGE**	**Aged over 60 years**	**1**
**BP**	**Hypertension (Systolic >140 and/or diastolic >90mmHg)**	**1**
**CLINICAL FEATURES**	**Unilateral weakness**	**2**
	**Speech disturbance without weakness**	**1**
**DURATION**	**Over 60 Mins**	**2**
	**10 – 59 mins**	**1**
**DIABETES**	**YES**	**1**

(High risk score = 4 or more. Low risk score = 3 or less). **Total ______**

What is the skill level of the person applying the ABCD2 score? Paramedic ☐ Technician ☐

STEP 3:

Consent

It is appropriate that you let your patient know that they are eligible for this study. **Please give your patient, or the nurse you hand over to, the TIA patient information envelope to be given to the patient before they leave hospital**; this contains information about the study and a consent form for them to sign and return if they would be happy to be phoned at 7 and 90 days. It is important to let them know that the treatment they receive will not be different, but the study will help us evaluate the ABCD2 score in the ambulance. It is also important that they travel to hospital with you. If present, and appropriate, it is advisable to bring any witness to the event along with the patient.

- Once at hospital, hand your patient over as per normal, including their ABCD2 score.
- **Please put this completed form in the envelope clearly marked for SECAmb TIA patients which should be positioned close to where you usually handover.**
- If your patient refuses to travel, document this as normal and please keep this form with your PCR to hand in at the end of the shift when you return to station.

Appendix B

Flow diagram for Surrey TIA patients

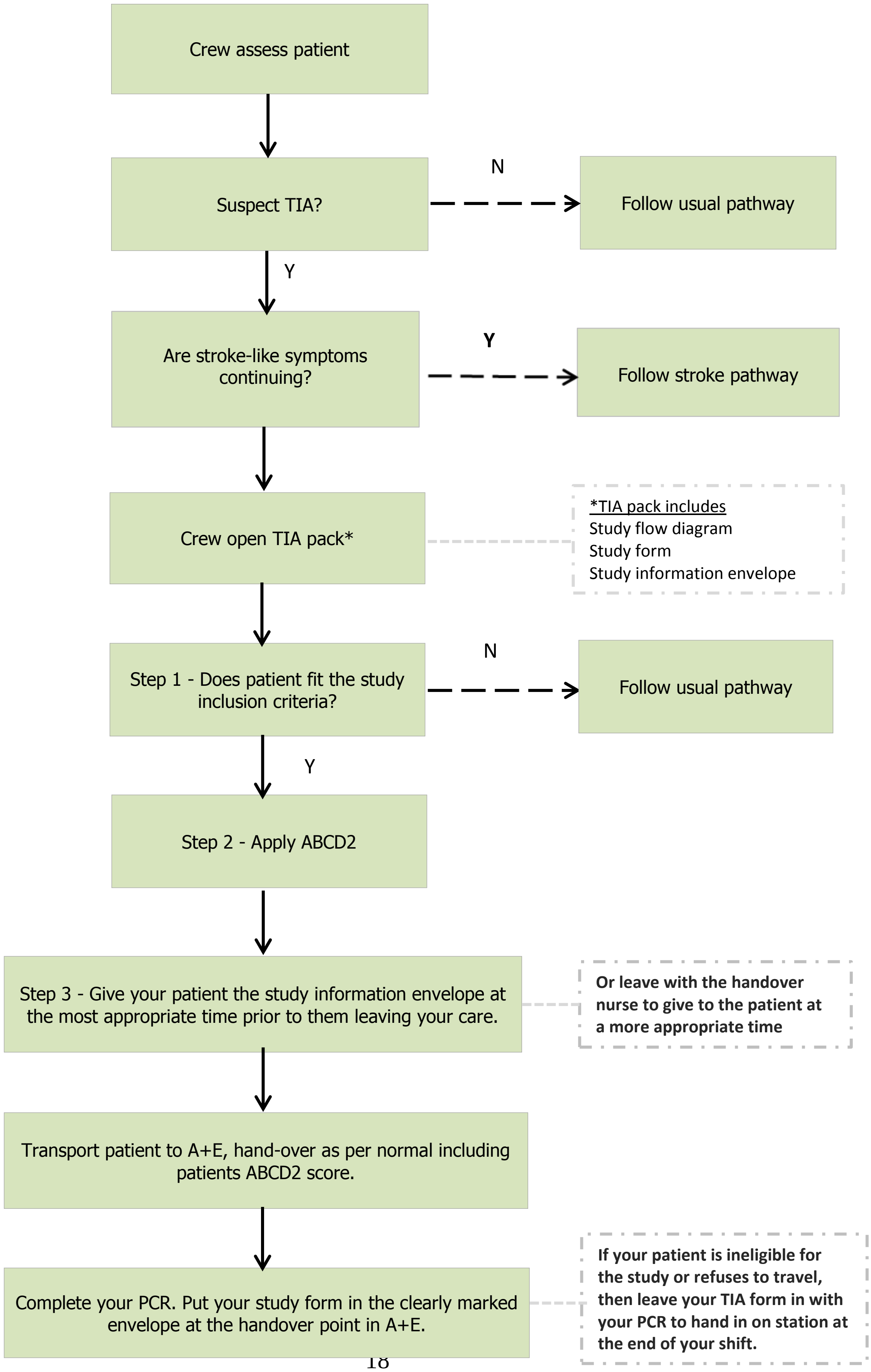

South East Coast Ambulance Service NHS
NHS Foundation Trust

Appendix C

TIA Study: Information for Participants

Version 3.6 Final 20 September 2012

Information about the research

We are giving you this information because you have been taken to hospital after having a suspected Transient Ischaemic Attack (TIA). A Transient Ischaemic Attack is a stroke-like event where symptoms last less than 24 hours.The Ambulance Service and the hospital that treats you are taking part in research that aims to improve how we assess and deal with cases like yours and this information sheet explains the study and what it means for you.

What is the purpose of the study?

We want very much to improve the quality of care and outcome for patients with suspected TIA. This study will help us to see whether ambulance staff can use a ‘risk score’ commonly used in hospital, and whether that score makes a difference for patients.

What exactly is being tested?

The ABCD2 Score is an assessment tool, commonly used in hospitals, that determines the patient’s risk of having a further TIA or stroke following a TIA and, as a result, informs the next course of action by hospital specialists. The score is based on Age, Blood pressure, Clinical presentation, Duration of symptoms and the presence of Diabetes, which are already collected by ambulance staff as part of normal patient assessment processes.

We want to learn if using the ABCD2 Score in the pre-hospital (ambulance) setting helps patients by speeding up hospital assessment and whether it accurately predicts the risk of a stroke.

Why have I been chosen?

You have been chosen because the ambulance staff think you have had a TIA, the patient group that the ABCD2 score is designed to be used to help.

Did I have to take part?

You do not have to take part in this study and of course we will continue to give you the best possible care whether you take part or not. Because of the urgency of the situation when you called the ambulance, it is not possible to ask people whether they want to take part before the ABCD2 score is calculated The Mental Capacity Act 2005 (Section 32, Para 104, subsections 8 & 9) allows for action to be taken in relation to the research where treatment is to be provided to the person urgently and there is insufficient opportunity to consult.

What will happen to me if I continue to take part?

If you agree to take part, a paramedic, nurse or doctor will contact you by telephone after approximately 7 days to ask if you would like to know more about the study before you decide whether or not we can include your results in the study. If you agree then you will be asked some questions about your health, and another telephone call will be made approximately 90 days later to ask you further questions about how you are.

What are the possible risks and benefits of taking part?
We do not anticipate there being any risks to you in this study.

What if something goes wrong?

It is extremely unlikely that anything will go wrong as a result of taking part in this study. However, if you feel that there has been negligence, then (as with any treatment) it is possible you may have grounds for a legal action against the relevant NHS Trust (Ambulance Service or Hospital), but you may have to pay your legal costs.

What happens if I have any questions, concerns or complaints about the study?

If you have any questions about the study or concerns about the way it has been carried out, you can tell the paramedic, nurse or doctor who contacts you, or you may contact the Ambulance Service direct (contact details below). They will do their best to help you. If you are unhappy about an aspect of your treatment and wish to complain formally, you can do this through the NHS Complaints Procedure.

Will my participation in the study be kept confidential?

Any information which is collected about you during the course of the study will be kept strictly confidential and will be seen only by authorised staff involved in the study and people from regulatory authorities who ensure that studies such as this one are carried out correctly. All of them have a duty of confidentiality to you as a research participant.

Information that is used in the study will include only what is needed in your medical records from South East Coast Ambulance Service (SECAmb) and from the hospital where you were treated. All information will be kept at a Study Coordinating Centre at SECAmb using secure methods that ensure that there will be no accidental disclosure of personal information.

The Study Coordinating Centre will need to keep records of your name and address and other contact details. Any information about you will be used only for this study and will not be given to anyone else. You have the right to see your personal health information related to the research study, but you will not be able to see some parts of the information until after the study has finished. When any information from the study is published it will not contain any personal information and it will not be possible to identify any individual.

The data from this study will be kept for at least 10 years after its conclusion and may be used in other research studies. If it is used in this way all personal identifiers will be removed and it will not be possible to identify any individual.

What will happen if I don’t want to carry on with the study?

You can tell us at any time if you want us to stop using your information in the study and of course we will continue to give you the best possible care whether you take part or not

What will happen to the results of the research study?

The study is expected to take at least one year. The study results will not be available until the autumn of 2013 or later. The results will be analysed and as well as presenting our findings at local and national meetings, we will submit them for publication in an appropriate medical journal. Our findings will also be available from the Ambulance Service and University of Surrey websites. When everything is finished we will offer you a copy of what we have learned.

Who is organising and funding the study?

The study is being organised by a group of nurses, paramedics, doctors and researchers from the NHS and the University of Surrey, led by Professor Tom Quinn.

Who has reviewed the study?

All research in the NHS is looked at by independent group of people, called a Research Ethics Committee, to protect your safety, rights, wellbeing and dignity. This study has been approved and given a favourable opinion by the NHS REC Committee (South East Coast), reference number 12/LO/1025, and by the University of Surrey Ethics Committee.

Contact details

South East Coast Ambulance Service NHS Foundation Trust:

Name: Sally Rodbard, Paramedic and TIA Study Project Manager
Address: Chertsey Ambulance Station
Guildford Road
Ottershaw, KT16 0PJ
Telephone number: 07717 130518
E-mail: sally.rodbard@secamb.nhs.uk

OR

Patient Advice and Liaison Service (PALS)
SECAmb Service NHS Foundation Trust
40-42 Friars Walk, Lewes, East Sussex, BN7 2XW
Tel: 01273 8978888

SECAmb website: www.secamb.nhs.uk

Chief Investigator: Professor Tom Quinn
Address: 23DK04, Duke of Kent Building
Faculty of Health and Medical Sciences
University of Surrey
Guildford, GU2 7XH
Telephone number: 01483 684553 or 01483 689319
E-mail: t.quinn@surrey.ac.uk

University of Surrey web site: www.surrey.ac.uk

South East Coast Ambulance Service NHS
NHS Foundation Trust

Evaluation of the ABCD2 Score in pre-hospital assessment of Patients with suspected Transient Ischaemic Attack (TIA): Pilot Study

South East Coast REC Ref No: 12/LO/1025

Appendix D

CONSENT FORM FOR PATIENT – CONFIDENTIAL

Version 3.6 FINAL 26 Sep 2012

	Please initial each box
1. I confirm that I have received a copy of, and have read and understood, the Patient Information Sheet Version 3.6 dated 20 September 2012 for this study, given to me by ambulance staff or hospital clinicians, and have had the opportunity to ask questions and discuss the study.	☐
2. I understand that my participation is voluntary and that I am free to withdraw at any time, without giving any reason, without my medical care or legal rights being affected.	☐
3. I confirm that I have had sufficient time to consider whether or not to continue in the study.	☐
4. I agree to relevant personal details being retained at the Study Coordinating Centre so that I can be contacted to complete the follow-up data collection.	☐
5. I understand that information held by the NHS and records maintained by The NHS Information Centre and the NHS Central Register may be used to help contact me and provide information about my health status.	☐
6. I agree to the telephone calls at approx 7 and 90 days collecting further information.	☐

Your preferred telephone number:

**Name of Patient**	**Signature**	**Date (dd/mm/yy)**

On completion, please return in the stamped addressed envelope provided.
